# Mechanistic Study of Hypoxia-Mediated Regulation of Osteoblast Senescence via ATP6V1A-Dependent Modulation of Metabolic Remodeling

**DOI:** 10.3390/biology14121801

**Published:** 2025-12-18

**Authors:** Hefang Xiao, Yi Chen, Xuening Liu, Rongjin Chen, Chenhui Yang, Fei Yang, Changshun Chen, Bin Geng, Yayi Xia

**Affiliations:** 1 Department of Orthopaedics, Lanzhou University Second Hospital, Lanzhou 730030, China; 120220901471@lzu.edu.cn (H.X.); yl15093051719@163.com (Y.C.);; 2 Gansu Province Orthopaedic Clinical Medicine Research Center, Lanzhou 730030, China; 3 Gansu Province Intelligent Orthopedics Industry Technology Center, Lanzhou 730030, China

**Keywords:** cellular senescence, hypoxia, osteoblast, ATP6V1A, metabolism, biomarkers

## Abstract

Background: Osteoblast senescence constitutes one of the major mechanisms in bone degeneration and is under tight regulation by metabolism and oxidative stress. While hypoxia has recently emerged as an important microenvironmental factor influencing the function of bone cells, its role in osteoblast senescence and metabolic regulation has yet to be defined. Methods: The present work entails hypoxia-modulated osteoblast senescence at one level, transcriptomic and metabolomic sequencing, and two levels, in vitro MC3T3-E1 and in vivo AAV-shAtp6v1a mouse models. In transcriptome profiling, hypoxia-responsive genes were identified, whereas non-targeted metabolomics was used to uncover metabolic alterations induced by ATP6V1A knockdown. Oxidative stress and mitochondrial function were assessed by qRT-PCR, Western blotting, SA-β-Gal staining, ROS detection, JC-1 mitochondrial potential, and immunofluorescence. Micro-CT, H&E, Masson, and immunohistochemistry studies were performed to investigate bone structure and protein expression in vivo. Results: Hypoxia markedly mitigated osteoblast senescence, decreasing p53 and p21 expressions and the number of SA-β-Gal-positive cells. It reduced intracellular ROS levels and increased HK2 and LDH expression, decreased ATP, and increased lactate, hinting at a shift toward glycolysis. Transcriptome analysis identified ATP6V1A as one of the major hypoxia-downregulated genes. Knockdown of ATP6V1A reduced ROS levels, inhibited p21 expression, improved mitochondrial function. Metabolomics disclosed remapping pathways in glycolysis, lipid, and amino acid metabolism. Conclusions: This study identifies a “Hypoxia–ATP6V1A–Oxidative Stress–Metabolic Remodeling–Anti-Senescence” axis, demonstrating that hypoxia delays osteoblast senescence by downregulating ATP6V1A, suppressing oxidative stress, and reprogramming metabolism, providing new insights and potential therapeutic targets for bone degenerative diseases.

## Simple Summary

Healthy bones rely on the activity of special bone-forming cells that gradually lose their strength and function as people age. This aging process contributes to osteoporosis and other bone-weakening conditions, yet the reasons why these cells age are still not fully understood. In our study, we explored how low-oxygen conditions, which naturally exist inside the body, influence the aging of bone-forming cells. We discovered that low oxygen reduces harmful molecules called reactive oxygen species, improves cellular energy balance, and slows the aging process of these cells. We also identified a protein called ATP6V1A that becomes less active under low-oxygen conditions and plays a central role in controlling stress and energy use inside the cells. When we reduced this protein in mice, their bone microstructural parameters improved, consistent with reduced cellular stress. Our findings reveal a previously unknown connection between oxygen levels, cellular stress, energy metabolism, and bone cell aging. This work may help guide the development of new strategies to maintain bone health and prevent age-related bone diseases in the future.

## 1. Introduction

Osteoporosis, a bone metabolic disease characterized by low bone mass and microarchitectural deterioration, still constitutes a major element of concern in the health of aging populations worldwide [1]. Inadequately, many cellular interactions and signaling pathways are involved in this complex pathology [2]. For instance, osteoblast dysfunction and senescence play an increasingly critical role in bone formation deficits and the increased severity of bone degenerative changes [3]. Under this condition, the senescence-associated p53/p21 and p16 pathways were reported, which seriously influence the life cycle of osteoblasts [4]. These pathways may be involved in the limitation of cell proliferation potential and also in the increased release of pro-inflammatory factors as senescence-associated secretory phenotype (SASP), resulting in further imbalance in bone remodeling [5,6]. In this respect, the highly hypoxic microenvironment that characterizes bone tissue strongly conditions the fate of cells and their metabolism. Conversely, under hypoxic conditions, HIFs represent the leading players in transcriptional regulation. They indeed balance the metabolic changes that occur within bone metabolism under conditions of oxygen deficiency [7]. Concomitantly, HIF-1α induces angiogenesis and glycolytic adaptation to hypoxia, while HIF-2α is a crucial regulator of lineage choice and bone resorption in marrow MSCs [8].

Hypoxia has thus been proposed as the crucial metabolic stress in the pathogenesis of osteoporosis [9]. In fact, in vitro experiments have shown that osteoblasts have low mitochondrial activity, increased reactive oxygen species (ROS) production, and energy metabolism disorders under chronic hypoxic conditions, which reduce the cells’ potential for differentiation and lead to senescence [10]. Moreover, in a series of recent studies, poor bone mineralization with increased osteoclastic activation through HIF-1α has been reported in the modeling of postmenopausal osteoporosis induced by estrogen-deficient conditions, suggesting a novel link in the pathogenic triad between hypoxic signaling and altered bone resorption in hormone imbalance [11,12]. At the same time, hypoxia signaling seems to regulate bone remodeling in several ways, to which resveratrol has been linked by several antioxidant mechanisms [13]. This decreases oxidative stress and therefore supports cellular homeostasis and bone remodeling balance while negatively influencing bone resorption [3]. This supports the view that both hypoxia and oxidative stress emerge in a concerted holdover of the regulatory network for cellular energetic metabolism required in the maintenance of bone homeostasis [14]. Indeed, recent literature has developed multilayered molecular hypotheses between the bone cell senescent state and the hypoxic-osteogenic microenvironment; nevertheless, some hypotheses, such as the senescence state through the p21 pathway, mitochondrial dysfunction, and ROS metabolism, are not well-developed at the level of the molecular mechanisms that can explain the involvement of hypoxia in osteoporosis [15]. To address this research gap, recent attention has shifted toward mitochondrial dysfunction and the role of ATP synthase subunits in bone metabolism [16]. The focus recently shifted toward mitochondrial disorders and, ATP synthase subunit assemblage in the metabolism of bone. Thus, the ATP synthase V1 subunit, contributing to energy production, is also possibly involved in the retention of the intracellular biological function of bone cells through senescence-related molecule expression regulation. A previous study supports the hypothesis that mitochondrial dysfunction affects the ability of oxidative phosphorylation in mesenchymal stem cells, NAD ATP, and consequently, cell differentiation, postulating that metabolic disorders as mechanisms brought about impaired bone tissue homeostasis [5]. From this line of evidence, the HIF–AMPK axis, which has been literally involved in mitochondria in relation to bone metabolism, indicates that AMPK, which is an energy sensor in the cell and responds to intracellular changes in the ATP/AMP ratio, is thus dependent on the activity of ATP synthase. This, in turn, will modify the signals coming via AMPK and control the destiny of osteoblasts under hypoxic conditions [17]. V-ATPase complex is distributed on cellular and intracellular membranes and maintains energy metabolism, lysosomal acidification, intracellular pH homeostasis, and signal transduction [18]. Recent studies have proposed that subunit a3 enhances proton secretion onto the resorptive surface and thus plays a functional role in resorptive activity by osteoclasts [19]. It has been suggested that abnormal V-ATPase activity disrupts proton pump function in osteoclasts, leading to excessive bone resorption and bone loss [20]. Moreover, normal V-ATPase activity is essential for metabolic crosstalk between lysosomes and mitochondria [21]. A blockade of this lysosomal proton pump precedes a loss of mitochondrial membrane potential and leads to an enhanced overproduction of ROS, disrupting energy homeostasis and activating the metabolism in bone cells [22]. Various subunits of the V-ATPase complex have been considered a variety of metabolic etiologies related to osteoblasts as well as marrow mesenchymal stem cells; therefore, they are interesting drug targets for bone-destructive disease [23]. Apart from that, scant knowledge exists on the V-ATPase complex complex and hypoxia-related osteoblast senescence and metabolic remodeling at the multi-omics level. Thus, an urgent need exists to synthesize information at the cellular, molecular, and animal levels to study the role of the hypoxic microenvironment in osteoblast senescence metabolism and deepen research on associated molecular targets of prevention and therapy in osteoporosis. In this study, we focus on how hypoxia regulates osteoblast senescence and oxidative stress and identify ATP6V1A as a key downstream mediator of this process. Using transcriptomic, metabolomic, and cellular analyses, we define a hypoxia–ATP6V1A–senescence axis that shapes osteoblast stress responses. The scope of this work is limited to senescence-related and metabolic alterations, and we do not evaluate osteoblast differentiation or osteogenic function. These boundaries clarify that the findings presented here relate to anti-senescence mechanisms rather than to bone formation outcomes.

## 2. Methods

### 2.1. Animal Experiments

All C57BL/6 female mice (20 weeks) were purchased from Lanzhou Veterinary Research Institute, Chinese Academy of Agricultural Sciences (Lanzhou, China), and were fed in a specific pathogen–free (SPF). The sample size was determined according to the principle of diminishing returns, meaning that increasing the number of animals beyond *n* = 6 would provide only marginal gains in statistical power while substantially increasing animal use and burden. Therefore, a sample size of six per group was selected to balance statistical reliability and ethical considerations. Afterward, bone tissues were collected after euthanasia for subsequent investigation. Twenty-week-old C57BL/6 mice were given randomly to two groups using a random number table: AAV9-Atp6v1a (*n* = 6) and AAV9-NC group (*n* = 6). Animals were raised under a 12 h intermittent light/dark cycle at 22 ± 2 °C with free access to chow and water. Fifty-two weeks after viral injection in vivo, mice from all groups were sacrificed, and serum and bones were collected. All animals/sections were serially and blindly numbered. All operations and interventions were approved by the Animal Ethics Committee of the Second Hospital of Lanzhou University (Approval No. D2023-350).

### 2.2. Cell Culture and Hypoxia Treatment

MC3T3-E1 cells are fetal mouse-derived osteoblastic cells provided by the Cell Bank of the Peking Union Medical College [24]. Cultured in α-MEM medium (Gibco, NY, USA) was supplemented with 10% fetal bovine serum (SE OU Biology, Shanghai, China) in a humidified atmosphere and 5% CO_2_ at 37 °C. Hypoxia treatment was conducted using a tri-gas incubator under hypoxic conditions consisting of 1% O_2_, 5% CO_2_ for 24 h. Control (normoxic condition) cells were grown under 21% oxygen. In addition, to directly evaluate the senescence response under oxidative stress, we established an osteoblastic senescence model by treating MC3T3-E1 cells with 200 μM H_2_O_2_ for 4 h. All in vitro experiments in this study were performed using the MC3T3-E1 pre-osteoblastic cell line (Cell Bank of Peking Union Medical College, Beijing, China).

### 2.3. Viral and siRNA Transfection

Lentiviral particles directed against the Atp6v1a gene were obtained from (GeneChem, Shanghai, China), and MC3T3-E1 cells were infected with the particles to obtain stable osteoblast cultures after puromycin screening. For phase-in vivo studies, bone-targeted adeno-associated virus (GeneChem, Shanghai, China) was intravenously delivered through the veins using tail injections. Viral titers were 1 × 10^12^ vg/mL.

### 2.4. Transcriptome Sequencing

To analyze the probable molecular mechanism of hypoxic intervention on osteoblasts, total RNA was extracted from MC3T3-E1 cells cultured in normoxia and hypoxia for 24 h by using Trizol method (Accurate Biology, Changsha, China), and its 260/280 ratio was detected for concentration and integrity, respectively. Separate libraries were constructed according to the instructions and used for PE-sequencing by the Illumina NovaSeq 6000 platform. Quality control checks of the original sequencing data used FastQC, version 0.11.9 and adapters and low-quality reads were removed to obtain clean reads. Clean reads were compared with the corresponding genome (mm^10^) using Hisat2 version 2.2.1, and transcript quantification was performed by FeatureCounts software version 2.0.1. Differential gene expression analysis was performed by Limma version 3.54.2 [25], differentially expressed genes were selected for enrichment analysis, including biological processes and pathways, such as Gene Ontology (GO) and Kyoto Encyclopedia of Genes and Genomes (KEGG) [26].

### 2.5. Metabolomic Profiling

For further investigation of the effect of Atp6v1a silencing on osteoblast metabolism, AAV-shAtp6v1a and AAV-NC samples were prepared and extracted with alcohol/water solution, homogenized, ultrasonically disrupted, centrifuged, and divided into positive and negative ion modes to profile the metabolites using LC-MS/MS (Thermo Scientific Q Exactive, Bremen, Germany). Compound Discoverer 3.1 was used for peak extraction, peak alignment, and peak area normalization. MetaboAnalyst version 5.0 was used for statistical analysis. Metabolites with a VIP score of >1 (obtained from OPLS-DA version 1.30.0) and a two-sided *p*-value < 0.05 were considered differential. A predictive model was constructed with a LASSO version 4.1-8 regression analysis method [27] and selected metabolites were further submitted to the KEGG pathway analysis to assess the diversity of metabolite categories and their associations with Atp6v1a.

### 2.6. Machine-Learning–Based Feature Selection for Transcriptomic and Metabolomic Datasets

To identify the most informative molecular features associated with hypoxia and Atp6v1a regulation, machine-learning approaches were applied to both the transcriptomic and metabolomic datasets. For transcriptome profiling, two complementary algorithms—Random Forest (RF) and Least Absolute Shrinkage and Selection Operator (LASSO) regression—were implemented to ensure robust feature selection. RF analysis was performed using the “randomForest” package in R with 500 decision trees, and gene importance was ranked according to the MeanDecreaseGini index. In parallel, LASSO regression was conducted with the “glmnet version 4.1-8” package, where 10-fold cross-validation was used to determine the optimal penalty parameter (λ·min). Genes with non-zero coefficients at λ·min were regarded as having strong predictive value under hypoxic conditions. For metabolomic profiling, LASSO regression was applied as the primary selection method because of the high dimensionality and intercorrelation of metabolites. The “glmnet version 4.1-8” package was used with cross-validation, and metabolites retaining non-zero coefficients at the optimal λ were considered candidate discriminative metabolites.

### 2.7. Measurement of ROS and Mitochondrial Membrane Potential

ROS production was measured using the fluorescent probe DCF-DA. Changes in mitochondrial membrane potential were assayed by JC-1 staining. Briefly, a 1 mL stock solution of JC-1 was diluted in 1 mL complete medium and incubated for 20 min at 37 °C followed by two washes with JC-1 buffer. Cells were then resuspended in 2 mL culture medium, and the changes in J-aggregated fluorescence compared to the monomeric form were observed by fluorescence microscopy (OLYMPUS, Tokyo, Japan).

### 2.8. SA-β-Gal Staining

According to the instructions of the senescence-associated β-galactosidase kit (Beyotime Biotechnology, Shanghai, China), cells were washed with PBS, fixed for 15 min, stained overnight with freshly prepared SA-β-Gal overnight at 37 °C, and observed under a microscope (EVOS XL Core, MA, USA).

### 2.9. RNA Extraction and qRT-PCR

Total RNA extraction was performed using Trizol reagent (Accurate Biology, Changsha, China). RNA was then reverse-transcribed by an Evo M-MLV RT PreMix Kit and quantified using the SYBR Green Pro Taq HS kit (Accurate Biology, Changsha, China) in a LightCycler 480 system (Roche, Basel, Swiss) with *GAPDH* as an internal control, the sequence of primers for target genes was showed in Supplementary Table S1.

### 2.10. Western Blot Analysis

Cells and tissues were lysed in RIPA buffer solution (Beyotime, Sshanghai, China), and protein concentrations were determined using BCA assay reagents (Beyotime, Shanghai, China). Proteins were electrophoretically separated by SDS-PAGE and transferred onto PVDF membranes (Millipore, MA, USA). Membranes were blocked with Protein Free Rapid Blocking Buffer (Epizyme, Shanghai, China) for 1 h at room temperature. Then, the PVDF membranes were incubated with the primary antibody at 4 °C overnight, namely, p53 Antibody (CST-2524T), ATP6V1A Antibody (Abcam-ab199326), p21 Antibody (proteintech-28248-1-AP), β-actin Antibody (proteintech-66009-1-Ig), HK2 (CST-C64G5), and LDH (AAbmart-T55348). The proteins were detected by an ECL kit (Biosharp, Hefei, China).

### 2.11. Immunofluorescence

The cells were washed three times with PBS and fixed in pre-cooled 4% paraformaldehyde for 30 min. After permeabilization with 0.1% Triton X-100 for 30 min at room temperature, it was blocked with 10% goat serum for 1 h at 37 °C. The cells were subsequently incubated with primary antibody overnight at 4 °C and a fluorochrome-conjugated species-specific secondary antibody for 1 h at 37 °C. After staining with DAPI, the cells were observed under a confocal laser scanning microscope (Carl Zeiss, Oberkochen, Germany).

### 2.12. Micro-CT and Histological Analysis

Each bone group will be fixed in 4% paraformaldehyde for 48 h, decalcified in 10% EDTA for 3 weeks, dehydrated in alcohol, embedded in paraffin, and cut into 5 μm slices. Scanco Viva CT 8.0 version 5.0 was used to assess BMD and BS/TV. H&E, Masson, and immunohistochemical staining were used to assess micro-architecture, collagen fibers, and senescence-related proteins in bone.

### 2.13. Statistical Analysis

Measurements from three independent experiments were averaged and expressed as mean ± SD; Student’s *t*-test or one-way analysis of variance was used for comparisons between the groups. Statistical significance was set at *p* < 0.05. All statistical analyses were performed using R 4.2.10 [28].

## 3. Results

### 3.1. Hypoxia Attenuates Osteoblast Senescence

**Figure 1:**
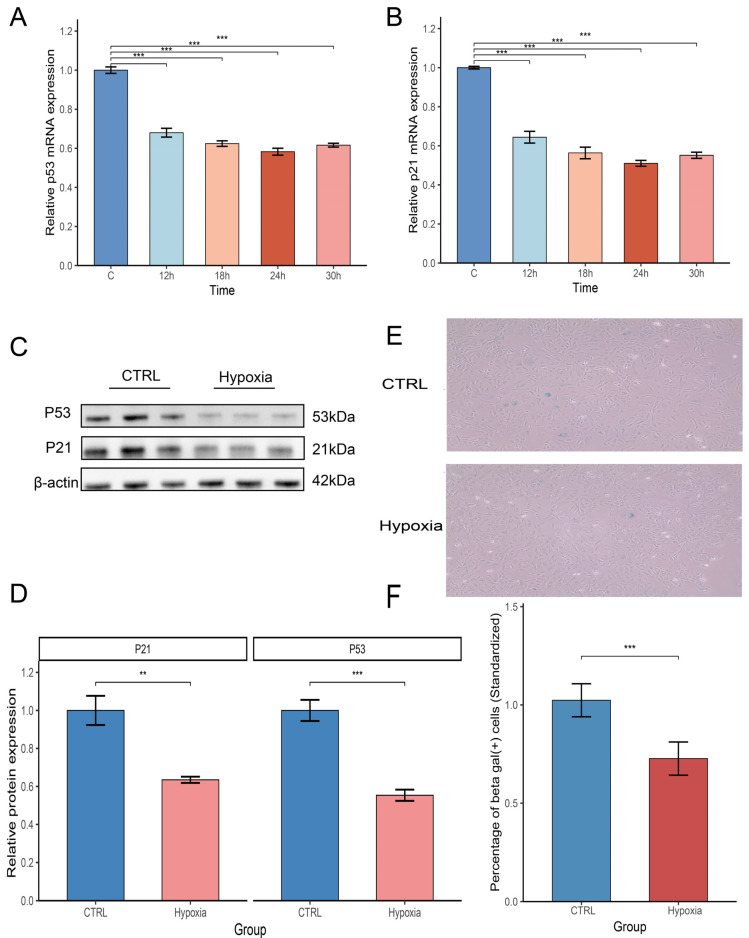
Effect of hypoxia on senescence of osteoblasts. (**A**,**B**) Expression levels of p53 and p21 mRNA relative to normoxia at different times of hypoxia treatment (*n* = 3). (**C**,**D**) Protein relative expression of p53 and p21 by Western blot assay (*n* = 3). (**E**) β-Gal staining to detect cell senescence (*n* = 3). (**F**) Quantification of SA-β-Gal-positive cells. ** *p* < 0.01, *** *p* < 0.001.

MC3T3-E1 cells were treated with hypoxia to detect the effect of hypoxia on cell senescence. The qRT-PCR results indicated that relative expression of mRNA in *p53* and *p21* was significantly lower than in the control group at 12 h of hypoxia (T1 group), and relative expression gradually decreased with extension of hypoxia time to 18 h (T2 group), 24 h (T3 group), and 30 h (T4 group)in a relative time-dependent manner (Figure 1A,B), which proved to be sample at the time of 24 h. Further Western blot analysis showed that p53 and p21 proteins were significantly lowly expressed in the hypoxia group when compared with the control group (Figure 1C), which was semi-quantitatively analyzed; (Figure 1D). In addition, sa-β-Gal staining indicated that the proportion of positive sa-β-Gal cells was reduced after hypoxia treatment (Figure 1E), i.e., the number of senescent cells in the hypoxia group was significantly lower than in the control group (*p* < 0.05) (Figure 1F).

### 3.2. Hypoxia Enhances the Metabolic Activity of Osteoblasts

**Figure 2:**
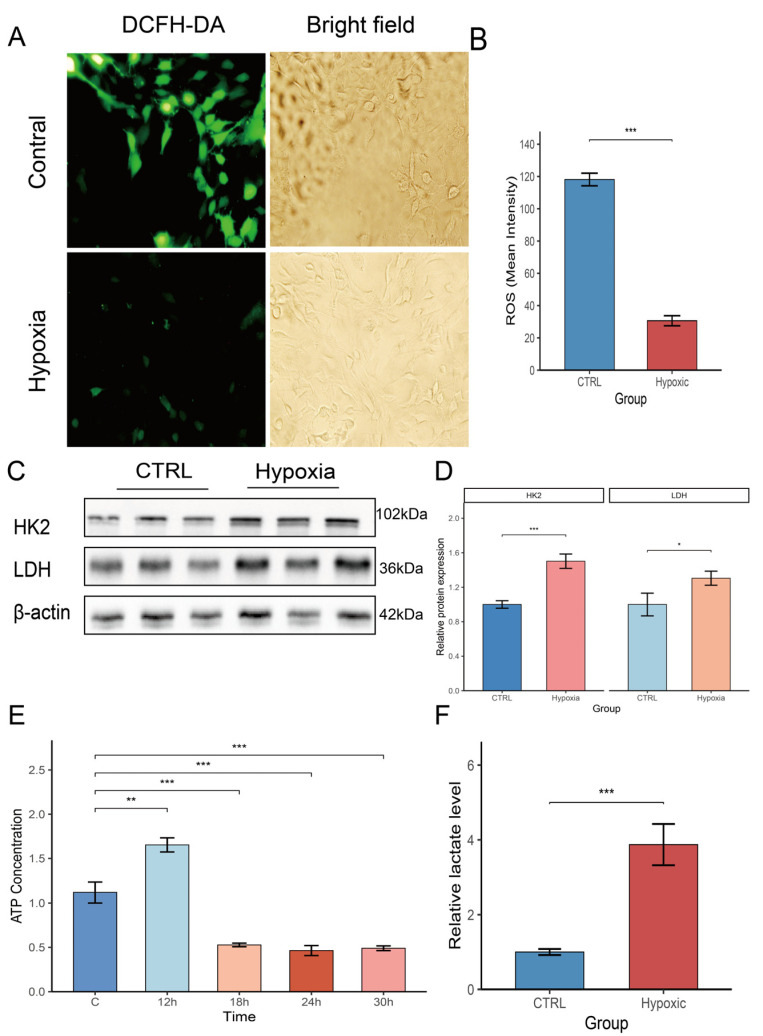
Hypoxia influences osteoblast metabolism. (**A**) DCF-DA for the detection of intracellular ROS. The fluorescent intensity of osteoblasts in the hypoxia group is much lower than that of the control group (*n* = 3). (**B**) Quantification of intracellular ROS production under different oxygen conditions: hypoxia restricts intracellular ROS production (*n* = 3). (**C**) Expression of HK2 and LDH in hypoxia and normoxic group cells by Western blot assay (*n* = 3). (**D**) Semi-quantitative analysis of protein expression levels using ImageJ software (version 1.54g) (*n* = 3). (**E**) Intracellular ATP levels detected under different oxygen conditions at different time points. Hypoxia reduces the intracellular ATP levels of osteoblasts within 24 h (*n* = 3). (**F**) Quantification of the assay for lactate (*n* = 3). * *p* < 0.05,** *p* < 0.01, *** *p* < 0.001.

Therefore, MC3T3-E1 cells were treated with hypoxia for 24 h to further elucidate the effects of hypoxia on metabolism. The generation of intracellular ROS under hypoxia was detected using DCF-DA fluorescent probes. In the hypoxia-treated group, ROS fluorescence was significantly inhibited compared with that in the control group (Figure 2A). These results quantitatively confirmed that hypoxia inhibited ROS production compared with that in the control group (Figure 2B). With respect to the metabolism-related proteins, Western blotting showed that HK2 and LDH in the hypoxia group were obviously increased compared with their levels in the control group (Figure 2C), which was also confirmed by semiquantitative analysis (Figure 2D). Then, ATP was detected at different time points. (Figure 2E) shows that ATP content in the hypoxia group was significantly decreased compared with that of the control group; the content decreased continuously with time, with the lowest value at 24 h. Lactate was also measured at 24 h, and there was a significant increase in the hypoxia group (*p* < 0.05) (Figure 2F). All the above results suggest that hypoxia changes the metabolism of osteoblasts toward glycolysis, decreases oxidative stress, and restructures the energy metabolism of osteoblasts under hypoxic conditions.

### 3.3. Identification of Key Genes Affected by Hypoxia

**Figure 3:**
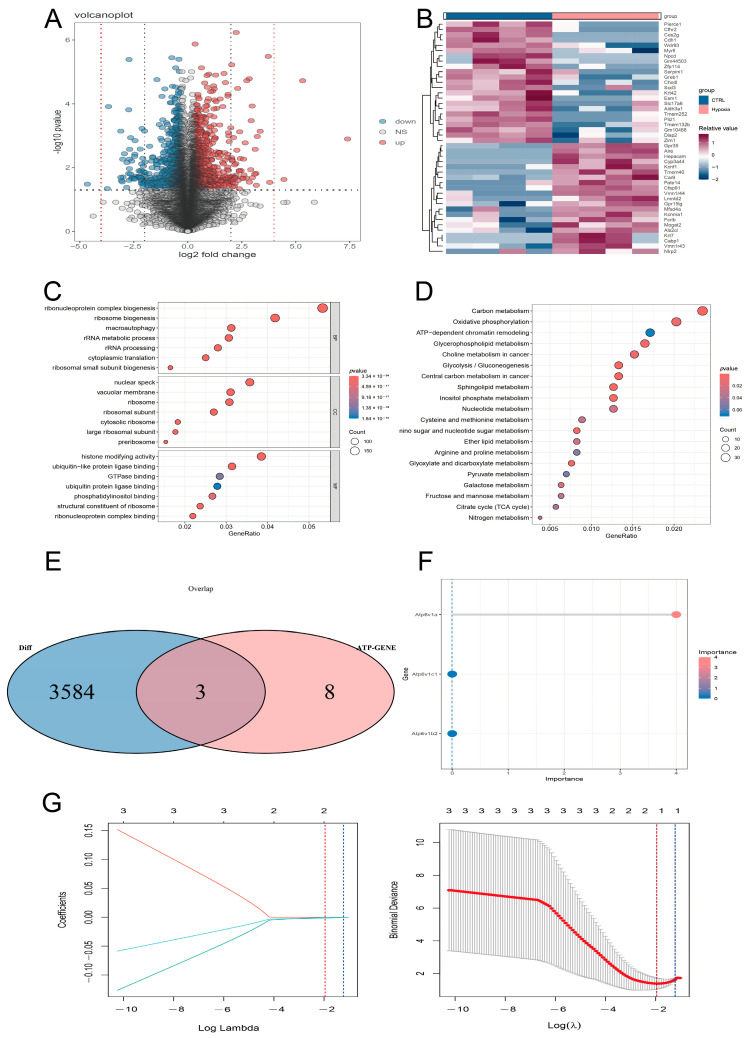
Transcriptomic analysis of hypoxia-induced osteoblast senescence: (**A**) Volcano plot of DEGs in two groups (*n* = 8). (**B**) Heat map for DEGs across two groups. (**C**) GO enrichment of DEGs. (**D**) Pathway analysis of KEGG. (**E**) Venn diagram of DEGs and ATP-V1 unit-related genes. (**F**) Generation of key genes by the random forest algorithm based on degree of importance. (**G**) Key genes generated by LASSO regression.

To better illustrate the molecular mechanism of hypoxia on MC3T3-E1 cells, transcriptome analysis of osteoblasts under hypoxia was performed, and the results were compared with those under normoxia. The volcano plot shows the number of DEGs found in the comparison group of hypoxia and control (Figure 3A). The heatmap illustrates that the DEG expression pattern under hypoxia is very different from that under normoxic conditions (Figure 3B). GO enrichment analysis indicates that the genes under the two conditions were mainly enriched in ribosome biogenesis, rRNA metabolic processes, cytoplasmic translation, and macroautophagy (Figure 3C). KEGG enrichment analysis indicates the metabolic signal pathway for carbon metabolism, glycolysis, gluconeogenesis, the tricarboxylic acid cycle, and oxidative phosphorylation in addition to KEGG (Figure 3D). The discovered bloom means that hypoxia may regulate the metabolic pathway of osteoblasts. We performed GSEA on the transcriptomic data, which showed that ATP6V1A-associated genes were mainly enriched in the c-type lectin receptor signaling pathway, the DAP12 signaling pathway, and FGFR4-related signaling pathways (Supplementary Figure S2).

Through previous literature and reviews, it was found that V1 subunit-related genes (Supplementary Tables S2) participated in metabolic progression, and to screen out the key genes in metabolic changes of osteoblasts in hypoxia, it was intersected with the screened DEGs (Supplementary Tables S3), and there were only three candidate key genes: ATP6V1A, ATP6V1C1, and ATP6V1B2 (Figure 3E). Secondly, using the random forest algorithm, the importance rankings of the three genes, related to the classification of the two groups, was obtained, and the first was ATP6V1A (Figure 3F). Finally, using the LASSO regression model to reveal the characteristic gene, it was found that ATP6V1A and ATP6V1B2 may be characteristic genes (Figure 3G). Referring to the above two machine learning methods, ATP6V1A was finally selected as the key metabolic gene of osteoblasts’ adaptation to hypoxia.

### 3.4. Validation of Atp6v1a Expression Under Hypoxic Conditions

**Figure 4:**
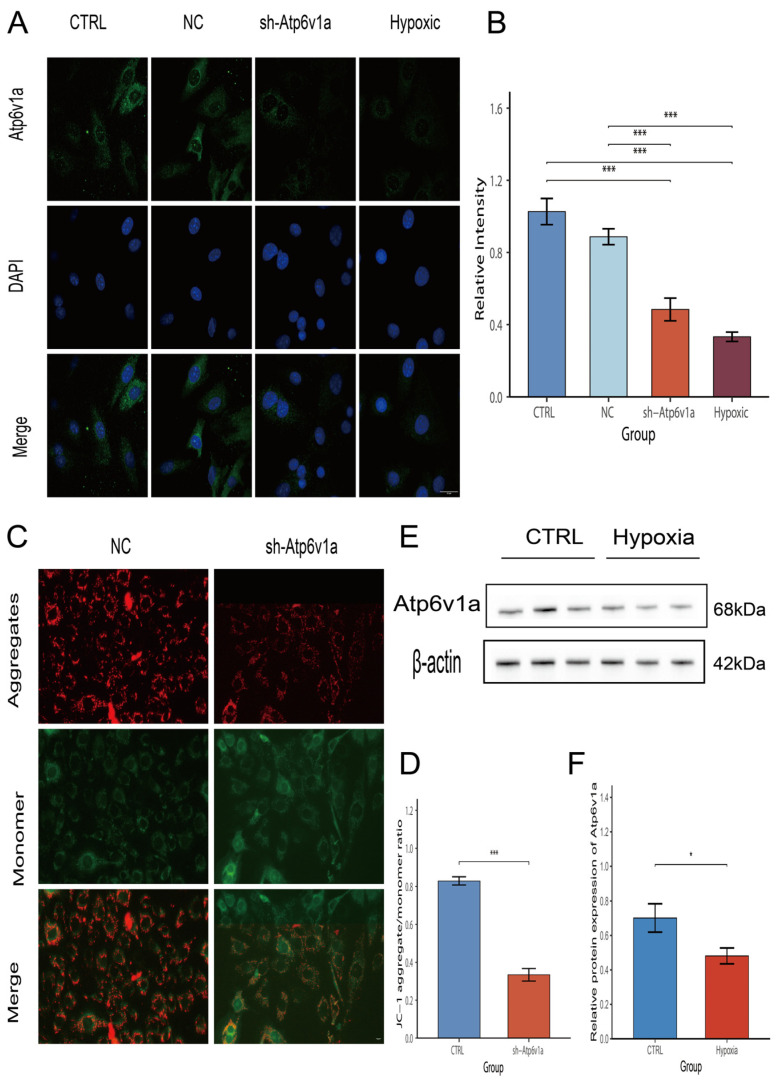
Expression of Atp6v1a in osteoblasts; the effect of its knockdown on mitochondrial function. (**A**): The confocal pictures of immunofluorescence presented the results that the signals of Atp6v1a were downregulated in the hypoxia and sh-Atp6v1a groups compared with the control group; bar = 5 μm (*n* = 3). (**B**): Quantitative fluorescence analysis showed a significant decline in relative Atp6v1a intensity. (**C**): JC-1 fluorescence staining showed that the red aggregated fluorescence of the sh-Atp6v1a group was decreased and green monomeric fluorescence was significantly increased; bar = 10 μm (*n* = 3). (**D**): Statistical results of red/green fluorescence showed a significant decline in mitochondrial membrane potential (*n* = 3). (**E**): Western blot showed that the protein expression of Atp6v1a was significantly downregulated under hypoxia (*n* = 3). (**F**): Semi-quantitation of Atp6v1a protein presented a significant decline (*n* = 3). * *p* < 0.05, *** *p* < 0.001.

We finally tried to confirm the expression of Atp6v1a in hypoxia-induced osteoblasts. We thus conducted a stable MC3T3-E1-shAtp6v1a cell line to explore the knockdown feature of Atp6v1a in hypoxic MC3T3-E1 cells. Further immunofluorescence confocal micrographs showed that the fluorescence of Atp6v1a was significantly lower in the hypoxia group and the sh-Atp6v1a group compared with the control group (Figure 4A). This finding was further confirmed by the significantly reduced average fluorescence intensity (*p* < 0.01). (Figure 4B). To confirm the efficiency of ATP6V1A knockdown, we performed validation experiments in vitro and in vivo. Western blot analysis demonstrated a marked reduction of ATP6V1A protein levels in MC3T3-E1 cells following lentiviral transduction (Supplementary Figure S1A,B). Consistently, immunofluorescence staining of femoral bone tissue from AAV-treated mice showed decreased ATP6V1A expression in vivo (Supplementary Figure S1C), qPCR analysis demonstrated that the ATP6V1A overexpression vector successfully increased ATP6V1A transcript levels in MC3T3-E1 cells (Supplementary Figure S1D), confirming effective gene silencing across both models.

JC-1 staining was then performed, showing that red aggregated fluorescence in the sh-Atp6v1a group was greatly reduced and green monomeric fluorescence was strongly expressed; thus, the ratio of red/green fluorescence was obviously decreased (Figure 4C,D), indicating that mitochondrial membrane potential was lost and mitochondria were damaged. This is consistent with Western blot showing that the protein expression level of Atp6v1a was significantly downregulated in hypoxia (Figure 4E) and semi-quantitative analysis of Western blotting, *p* < 0.05 (Figure 4F). All these results confirmed the transcriptional results; therefore, Atp6v1a is one of the active regulatory molecules of osteoblasts under hypoxic conditions.

### 3.5. Effects of Atp6v1a Knockdown on Osteoblast Senescence

**Figure 5:**
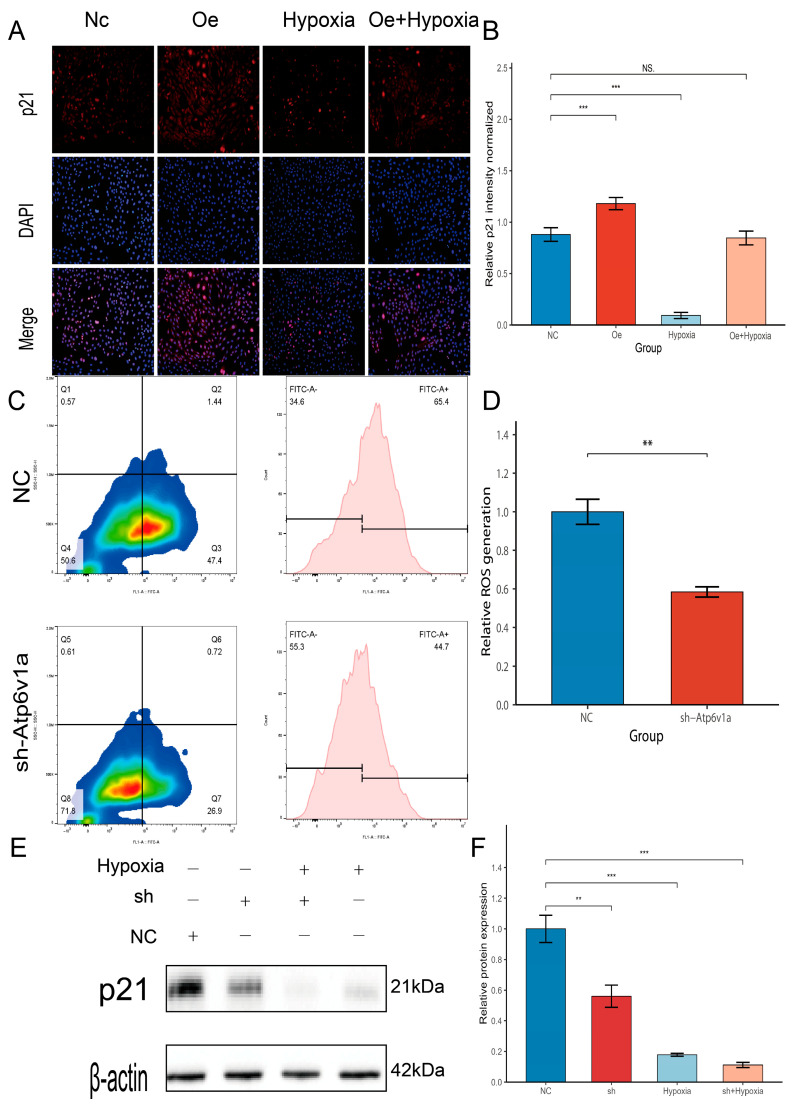
The role of Atp6v1a in silencing osteoblast senescence. (**A**): Immunofluorescence of p21 showed that the fluorescence was increased in the Oe Atp6v1a group, decreased in Hypoxia, and partially rescued under the Oe Atp6v1a + Hypoxia condition; bar = 10 μm (*n* = 3). (**B**): Statistical analysis of p21 fluorescence intensity. (**C**): Intracellular ROS levels by flow cytometry: (**D**): Sh-Atp6v1a inhibits the generation of ROS. (**E**,**F**): Western blot of β-actin, p21, and statistical analysis (*n* = 3). ** *p* < 0.01, *** *p* < 0.001, NS = not significant.

To further confirm whether osteoblast senescence was related to the change of Atp6v1a, corresponding intervention experiments were carried out to detect the expression of p21 at this time point. Western blot results showed that p21 protein expression was downregulated in the sh-Atp6v1a group, a trend also induced by hypoxia, and knockdown combined with hypoxia resulted in the lowest expression of p21 protein (Figure 5E), and statistical semi-quantitative analysis showed significant declines (Figure 5F). Then, rescuing experiments were performed. Immunofluorescence experiments showed that p21 fluorescence intensity was obviously upregulated in the oe-Atp6v1a group, downregulated in the hypoxia group, and, correspondingly, in the overexpression group combined with hypoxia, partly upregulated compared with the single overexpression intervention (Figure 5A). Quantitative fluorescence analysis validated this speculation: Atp6v1a knockdown did reduce the expression of p21 in terms of fluorescence intensity (Figure 5B). Further, flow cytometry using the DCF-DA (2′,7′-dichlorodihydrofluorescein diacetate) probe showed that intracellular ROS levels were markedly reduced in the sh-Atp6v1a group (Figure 5C,D). Altogether, sh-Atp6v1a reduced ROS content and the senescence relayed protein p21, effectively blunting the senescent phenotype of osteoblasts. Supplementary Figure S3 further validates the role of ATP6V1A in modulating oxidative-stress–induced senescence in osteoblastic cells. As shown in Supplementary Figure S3A, SA-β-gal staining revealed a marked increase in senescent cells following H_2_O_2_ exposure, whereas co-treatment with sh-Atp6v1a visibly reduced the proportion of SA-β-gal–positive cells. Quantitative analysis (Supplementary Figure S3B) confirmed these observations, demonstrating significantly elevated senescence levels in the H_2_O_2_ group and a clear attenuation of senescence in the H_2_O_2_ + sh-Atp6v1a group. Consistent with these findings, p21 immunofluorescence staining (Supplementary Figure S3C) showed robust induction of p21 upon H_2_O_2_ treatment, while ATP6V1A knockdown partially reversed this increase. Quantification of fluorescence intensity (Supplementary Figure S3D) reproduced the same trend, indicating that ATP6V1A silencing mitigates H_2_O_2_-induced activation of senescence markers. Similarly, qPCR analysis of p21 mRNA (Supplementary Figure S3E) showed a pronounced elevation in the H_2_O_2_ group and a significant decrease following ATP6V1A knockdown.

To further examine potential effects on extracellular matrix–related gene expression, we assessed COL1A1 transcript levels. As shown in Supplementary Figure S1F, ATP6V1A knockdown resulted in a mild but consistent upregulation of COL1A1, which aligns with the improved collagen organization observed in vivo. The original Western blot images have been uploaded as Supplementary Material and are now included as Supplementary Figure S4.

### 3.6. Metabolomic Analysis Following Knockdown of the Key Gene

**Figure 6:**
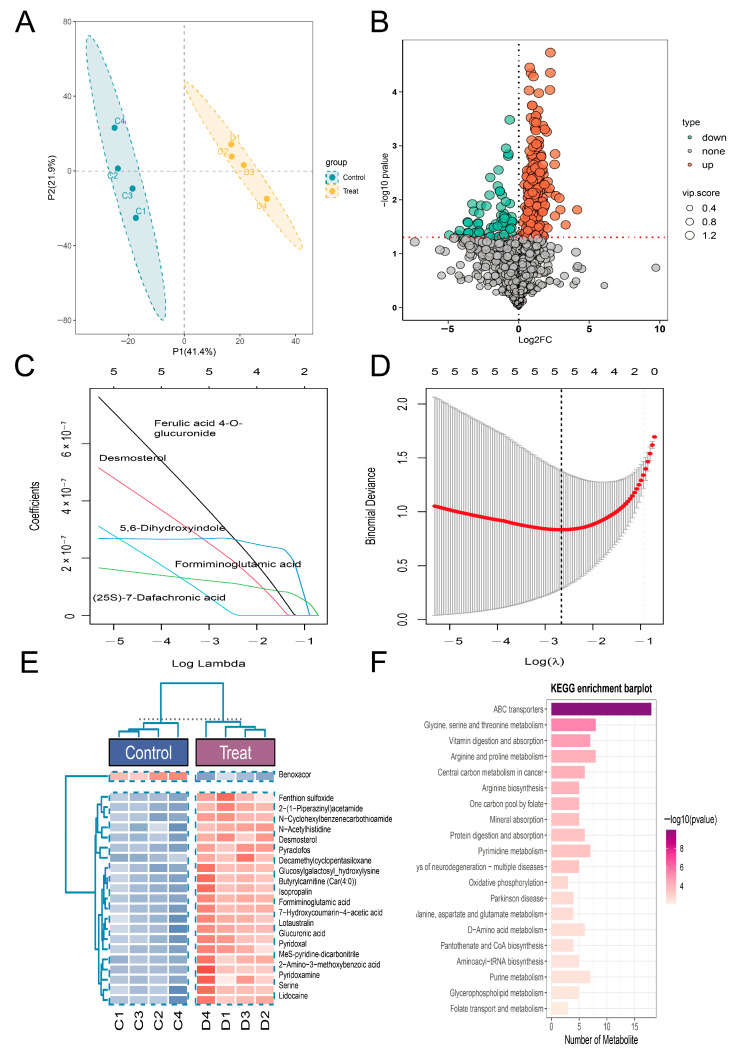
Metabolomics analysis of Atp6v1a knockdown. (**A**): PCA score plot, *n* = 8; (**B**): Volcano map based on differential metabolites; (**C**,**D**): LASSO regression screening of differential metabolites; The red dot indicates the optimal feature selected by the model, and the dashed line represents the corresponding optimal parameter; (**E**): Heat map of differential metabolites between different groups; (**F**): Metabolomics KEGG enrichment histogram.

Undoubtedly, to understand the mechanism of Atp6v1a regulation of osteoblast metabolism, we applied non-targeted metabolomics in Atp6v1a-knocked osteoblasts and controls. The PCA plot revealed that sh-Atp6v1a caused significant metabolic changes in the osteoblasts (Figure 6A). According to the character of the volcano plot shown in Figure 6B, Atp6v1a knockdown induced the modification of various metabolites up or down, thus confirming the command to search for typical differential metabolites using LASSO regression modeling. After many attempts, five candidate metabolites were finally identified: Ferulic acid 4-O-glucuronide, Desmosterol, 5,6-Dihydroxyindole, Formiminoglutamic acid, and (25S)-7-Dafachronic acid (Figure 6C,D). Expression abundances were different in osteoblasts with or without Atp6v1a knockdown as displayed by the heat map (Figure 6E). KEGG study showed mainly amino acid metabolism, glucolipid metabolism, purine metabolism, lipid metabolism, and vitamin absorption (Figure 6F).

### 3.7. In Vivo Validation of Atp6v1a Function

**Figure 7:**
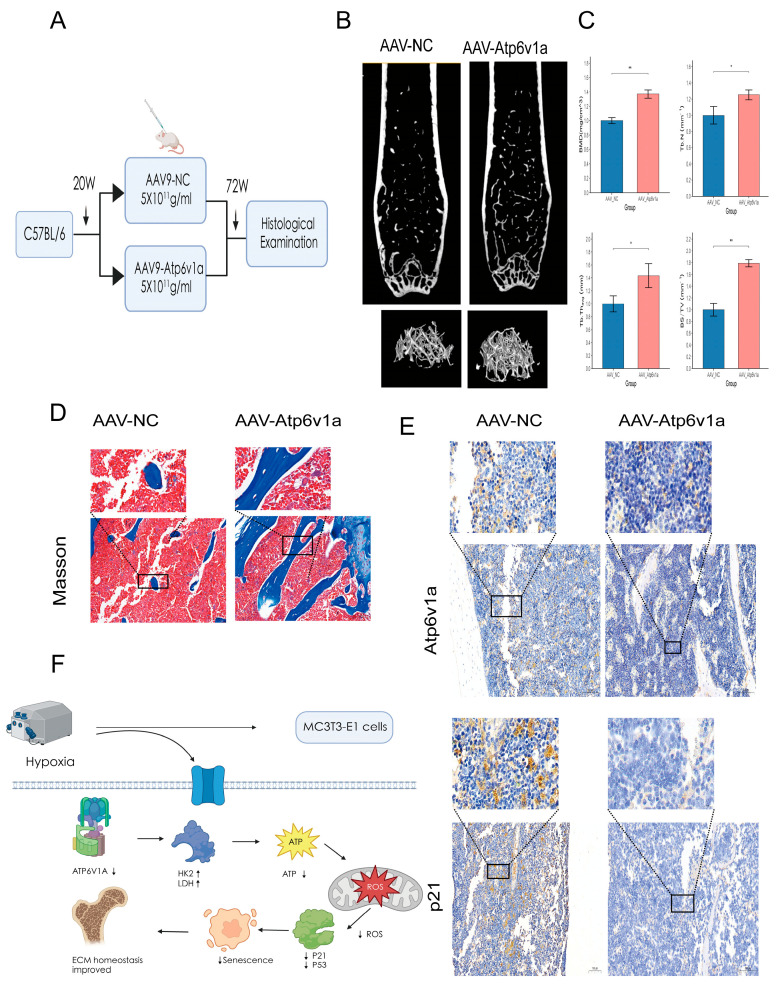
The representative in vivo assay of Atp6v1a silencing on bone tissue and the possible mechanism model. (**A**) A schematic diagram of the experimental design: Twenty-week-old C57BL/6 mice were injected with AAV-shAtp6v1a or AAV-NC through the tail vein and sacrificed 52 weeks after injection. (**B**) Representative micro-CT 3D reconstruction images demonstrating improved bone structure in the femurs of mice in the AAV-shAtp6v1a group compared to the AAV-NC group (*n* = 6). (**C**) Quantitative micro-CT analysis showed significantly increased BMD, BS/TV Tb. Th and Tb. N in the AAV-shAtp6v1a group (*n* = 6). (**D**) Masson staining showed that collagen fiber deposition was increased, and bone matrix was dense in the AAV-shAtp6v1a group (*n* = 6). (**E**) IHC showed reduced ATP6V1A and p21 expression levels in AAV-shAtp6v1a (*n* = 6). (**F**) Schematic model: Hypoxia inhibits ATP6V1A to reduce ROS production and causes metabolic switching to glycolysis-dominant metabolism. * *p* < 0.05, ** *p* < 0.01.

Subsequently, after hypoxia stimulation in vitro, we further studied its regulatory role in vivo by establishing a mouse femur model of AAV-shAtp6v1a intervention (Figure 7A). Micro-CT showed changes in trabecular microstructural parameters, with higher BMD values observed in the AAV-shAtp6v1a group. These structural differences are consistent with reduced cellular senescence (Figure 7B). Quantitative analysis showed that the AAV-shAtp6v1a group had a significant increase in BMD and BV/TV compared with the AAV-NC group (Figure 7C). Masson staining showed more organized collagen fibers and a denser matrix architecture, consistent with improved ECM structural maintenance.in AAV-shAtp6v1a (Figure 7D). Moreover, immunohistochemical staining showed an obvious decrease in ATP6V1A and its downstream target, senescence-related protein p21, in AAV-shAtp6v1a (Figure 7E). According to the above evidence, we present a mechanistic theoretical model: hypoxia, through inhibition of ATP6V1A expression, reduces oxidative stress and activates glycolysis to inhibit ROS production to mitigate osteoblast senescence and bone structural changes (Figure 7F).

## 4. Discussion

Osteoporosis is a typical age-related bone metabolic disease, which is presented as low BMD, trabecular microstructure damage, and an increase in fracture cutoff [29]. In this regard, osteoporosis is rapidly developing as a global public health problem and socio-economic burden with aging [30]. Osteoblast dysfunction and senescence have widely been discussed as mechanisms underlying osteoporosis development and progression [31]. Osteoblasts are known to be bone-forming cells that secrete bone matrix proteins and coordinate bone matrix mineralization in bone homeostasis. In the physiology of aging and pathological stress, osteoblast cellular senescence occurs, causing defective proliferation, secretory dysfunction, and cell cycle interference [32]. The senescence of osteoblasts is related to increased expression of senescence markers p53 and p21, which are activated in response to DNA damage, oxidative stress, and inflammation, and exert a central regulatory role in the maintenance of cellular homeostasis [33].

The phenotypic changes observed in this study primarily reflect senescence-related alterations. In this regard, mitochondrial dysfunction and oxidative stress may result in osteoblast senescence, as high ROS levels can develop oxidative DNA damage, which causes premature senescence related to mitochondrial dysfunction [3]. Mild hypoxia is one of the potential inducers responsible for pathological alterations in osteoblast cell function in senility, which would be interesting for hypoxia-related investigation due to a fairly restricted blood supply to the bone. Recently, the HIF-signaling pathway has been suggested to have systemic regulation in bone physiology. The role of HIF in hypoxia-mediated transactivation of VEGF and EPO under low oxygen tension has been causatively linked to the attainment of angiogenesis and bone homeostasis. These solid linkages between hypoxia research in therapeutic interventions targeting osteoblast senescence and bone matrix homeostasis. On the other hand, oxidative stress in osteoblasts may also participate in ferroptosis and apoptosis, thus even aggravating skeletal deterioration. Therefore, Liu et al. developed a phototriggered CO-releasing system for the activation of ferritinophagy in tumor cells metastatic to the bone, which inhibits tumor growth by ferroptosis [34]. The other study reviewed the oxygen-sensing mechanism of osteocytes under conditions of oxygen deprivation in osteoporosis and pointed out that osteocytes are sensitive to levels of local oxygen tension, upon which metabolic or transcriptional adaptation is reshaped via hypoxia signaling [35]. Overall, the disease mechanism and treatment of antioxidant balance in bone are under oscillated attention. Further research needs to be performed on balancing ROS levels and exploring selective antioxidant therapies to maintain bone homeostasis.

Therefore, a developing axis of “hypoxia-osteoblast senescence-metabolic rewiring” has been formed; hence, much bearing multieffects of hypoxia on osteoblast senescence and metabolic remodeling is systematically evidenced, with highly relevant ATP6V1A high-up to such axis. A moderate hypoxic environment did noticeably reduce the aging phenotype of osteoblasts upon reduced relative mRNA levels and expressed proteins p53, p21, and a much lower number of SA-β-Gal–positive cells, suggesting that under hypoxia, intracellular stress is limited and intracellular homeostasis is regained, indicative of a delay in the aging process of osteoblasts. Compared with normoxia, hypoxia modulated the metabolism of osteoblasts in a differential manner. ROS analysis revealed hypoxia to reduce intracellular ROS levels, cohering with up-regulation of HK2 and LDH, glycolytic enzymes. Moreover, intracellular ATP was found to be decreased, while intracellular lactate increased, suggesting that in hypoxic conditions, the cells switch their metabolism toward glycolysis-altered pathways. Metabolically, this provides cells with ATP compared to normoxia while reducing mitochondrial oxygen consumption through the electron transport chain; thereby, efficient hypoxia limits intracellular ROS production. Supporting this assumption, hypoxia has affected gene expression profiles in several metabolic and cellular homeostasis pathways, most strikingly in carbon metabolism, the TCA cycle, and oxidative phosphorylation.

After intersecting DEGs and ATP-V1 subunit genes, using Rand Forest analysis combined with Lasso regression, ATP6V1A was screened out as a highly prioritized hypoxia-related gene. Repressed ATP6V1A, then recognized metabolically rewired and antioxidant-related, was further functionally verified: knockdown of this gene strongly suppressed p21 and ROS levels and reduced the mitochondrial membrane potential measured by JC-1 staining, a decline indicating mitochondrial dysfunction, along with diminished senescence. Further metabolomics profiling posited the above postulates, Isocitric acid, 4-(2′-propenyl)-2-O-glucuronide, desmosterol, and formiminoglutamic acid are unique biomarkers of metabolism modulated by Atp6v1a. This has elucidated Atp6v1a independent regulation of energy and material homeostasis of osteoblasts via amino acids, glycolysis/gluconeogenesis, lipid, and purine metabolism. This was finally supported by in vivo evidence that Micro-CT revealed improved bone microstructural parameters, which are plausibly attributable to reduced cellular senescence and improved ECM maintenance rather than changes in osteogenic differentiation.; immunohistochemistry revealed reduced simultaneous expression of Atp6v1a and downstream senescence protein p21, and Masson and H&E staining identified more organized collagen deposition and denser matrix architecture. Above all, the “hypoxia–ATP6V1A–metabolic reprogramming–anti-aging” pathway was identified and verified, through which decreased ATP synthesis inhibits intracellular ROS generation and mitochondrial oxidative stress, thus retarding osteoblast senescence. While much of the research has focused on hypoxia and inflammation, few authors have highlighted the influence of hypoxia on aging and energy metabolism in osteoblasts. Indeed, for instance, Zhang et al. reported that RORβ deletion promotes endochondral ossification by regulating the HIF-1α/VEGFA signaling pathway [36]. Yang et al. have reviewed the energy sensing-regulatory function of the V-ATPase in jaw necrosis and periodontal lesions. However, these investigations of acidification of osteoclasts concentrated on the crosstalk between hypoxia and osteoblastogenic metabolic processes [37]. Qiu et al. also reported that a3 subunit knockout results in AMPK-mTOR-autophagy-dependent subchondral bone osteoarthritis [38], osteoclast-related processes rather than osteogenic regulation. For the first time, this study identified ATP6V1A as hypoxia-responding in osteoblasts, which is closely related to intracellular ROS levels, mitochondrial membrane potential, and p16 expression. The current study thus provides evidence for a cross-lineage regulatory role of V-ATPase subunits in bone remodeling, from oxidative stress to metabolism and senescence. Previous studies have clearly shown that osteoblasts entering a senescent state undergo intrinsic functional decline, including reduced extracellular matrix (ECM) secretion, impaired collagen production, and diminished mineralization capacity. These alterations disrupt bone matrix homeostasis and ultimately lead to a progressive decrease in bone mass and quality [39,40]. These findings indicate that senescence-driven impairment of ECM maintenance itself can influence bone tissue integrity. In our study, we observed a mild upregulation of COL1A1 expression following shATP6V1A treatment, a trend consistent with the denser and more organized collagen fibers observed in Masson staining in vivo. These results suggest that reducing cellular senescence may help restore ECM structural integrity, thereby contributing to improved tissue-level bone homeostasis.

In this study, the observed decrease in mitochondrial membrane potential (ΔΨm) and the reduction in ROS levels following ATP6V1A downregulation indicate a “compensatory and protective” metabolic adaptation. Under conditions of acute hypoxia and energy stress, cells can moderately lower ΔΨm to reduce the burden on the electron transport chain, thereby limiting electron leakage and decreasing oxidative stress. Previous research has shown that such regulated decreases in membrane potential are an important mechanism for maintaining mitochondrial stability during hypoxic adaptation, effectively reducing oxidative pressure while preserving mitochondrial function in adverse environments [41]. In addition, Schiffer et al. demonstrated that “mild mitochondrial uncoupling” exerts clear tissue-protective effects by lowering membrane potential to reduce ROS production without impairing cellular metabolic capacity [42]. Taken together, the ΔΨm reduction and ROS decrease observed after ATP6V1A silencing in our study represent a protective metabolic reprogramming under hypoxic and energy-limited conditions, consistent with previously reported mechanisms. In addition, when glycolysis is enhanced, V-ATPase activity tends to increase, whereas inhibition or disassembly of V-ATPase forces the cell to rely more heavily on glycolysis. This indicates that V-ATPase, including its ATP6V1A subunit, is closely coupled with glycolytic flux rather than functioning independently [43]. Moreover, under hypoxic conditions, cells shift their metabolic strategy from mitochondrial oxidative phosphorylation to a glycolysis-dominant mode to maintain ATP production [44]. Integrating these findings with our own results, we propose a mechanistic scenario in which ATP6V1A downregulation leads to reduced V-ATPase functionality, resulting in impaired proton pump activity and disrupted maintenance of ion gradients and endosomal–lysosomal acidification, which collectively limit mitochondrial respiration and overall cellular energy metabolism. In response to this energy-restricted state, osteoblasts upregulate glycolytic enzymes such as HK2, LDHA, and PGK, accompanied by increased lactate production, reflecting a compensatory enhancement of glycolysis to sustain cellular energy homeostasis. According to previous studies, acute hypoxia and chronic hypoxia activate fundamentally different cellular programs. Acute hypoxia generally triggers compensatory, adaptive, and protective responses rather than cytotoxic effects. In the early stage of acute hypoxia, HIF-1α is rapidly stabilized and initiates metabolic adaptation, including the up-regulation of key glycolytic enzymes such as HK2 and LDHA. This metabolic switch from mitochondrial oxidative phosphorylation to glycolysis reduces oxygen consumption, limits electron leakage, and suppresses ROS generation [45]. In addition, hypoxia-activated HIF-1α signaling has been shown to decrease oxidative stress, maintain mitochondrial homeostasis, and prevent oxidative damage within an appropriate range [46]. Therefore, short-term hypoxia exerts protective effects through metabolic reprogramming and ROS suppression, whereas substantial cellular injury is more characteristic of sustained, chronic hypoxia. The reduced ROS levels and alleviated senescence phenotypes observed in this study are consistent with these well-established acute hypoxic adaptive mechanisms.

The present study has several limitations. First, the conclusions have not yet been directly validated in human bone tissues, and the clinical translational value requires further clarification. Second, while ATP6V1A is identified as a critical regulator of hypoxia-related senescence, its extended signaling interactions and long-term effects on skeletal homeostasis remain important subjects for future study. Third, because osteoblast senescence and osteogenic differentiation represent biologically distinct processes, and senescence alone can impair bone matrix maintenance, this study did not include functional assays of osteogenic differentiation. These experiments, including standard induction and mineralization assays to assess the potential effects of ATP6V1A, will be systematically investigated in future work. In addition, we plan to employ second harmonic generation (SHG) imaging to analyze collagen and bone matrix structural alterations at higher resolution. In addition, we will further verify the role of the AMPK signaling axis and other energy-sensing pathways to more fully clarify the mechanism by which ATP6V1A regulates osteoblast metabolism and aging.

## 5. Conclusions

In summary, a series of in-depth studies on the role of hypoxia in osteoblast senescence and metabolic remodeling highlighted ATP6V1A as one of the essential molecular protagonists. This even proved that mild hypoxia delays osteoblast senescence by reducing ROS accumulation, inhibiting the p53/p21 signaling pathway, and switching cellular metabolism predominantly to glycolysis. Multi-omics analysis confirmed that ATP6V1A is a core hub in hypoxia-induced metabolic remodeling. Thus, the present work identifies the novel axis “hypoxia-ATP6V1A-metabolic reprogramming-anti-aging” and offers further mechanistic insights into bone aging and additional potential intervention targets for metabolic and oxidative stress-related degenerative diseases.

## Data Availability

Data will be made available on request from the corresponding author.

## Supplementary Materials

The following supporting information can be downloaded at: https://www.mdpi.com/article/10.3390/biology14121801/s1. Figure S1: Validation of ATP6V1A knockdown and overexpression in vitro and in vivo; Figure S2: Gene Set Enrichment Analysis (GSEA) of ATP6V1A-associated transcriptional programs; Figure S3: Validation of ATP6V1A knockdown in an H_2_O_2_ induced senescence model; Figure S4: Supplementary information–uncropped immunoblots; Table S1: Primers used for qPCR; Table S2: V1 subunit–related genes; Table S3: Differentially expressed genes (DEGs) identified.
