# Supplementary material for: Mechanistic Study of Hypoxia-Mediated Regulation of Osteoblast Senescence via ATP6V1A-Dependent Modulation of Metabolic Remodeling
Source: Biology (Basel). 2025 Dec 18;14(12):1801. doi: 10.3390/biology14121801 (PMC12731071; doi:10.3390/biology14121801)
Supplement: Supplementary file 1 [file biology-14-01801-s001.zip › Supplementary Figure2.pdf]

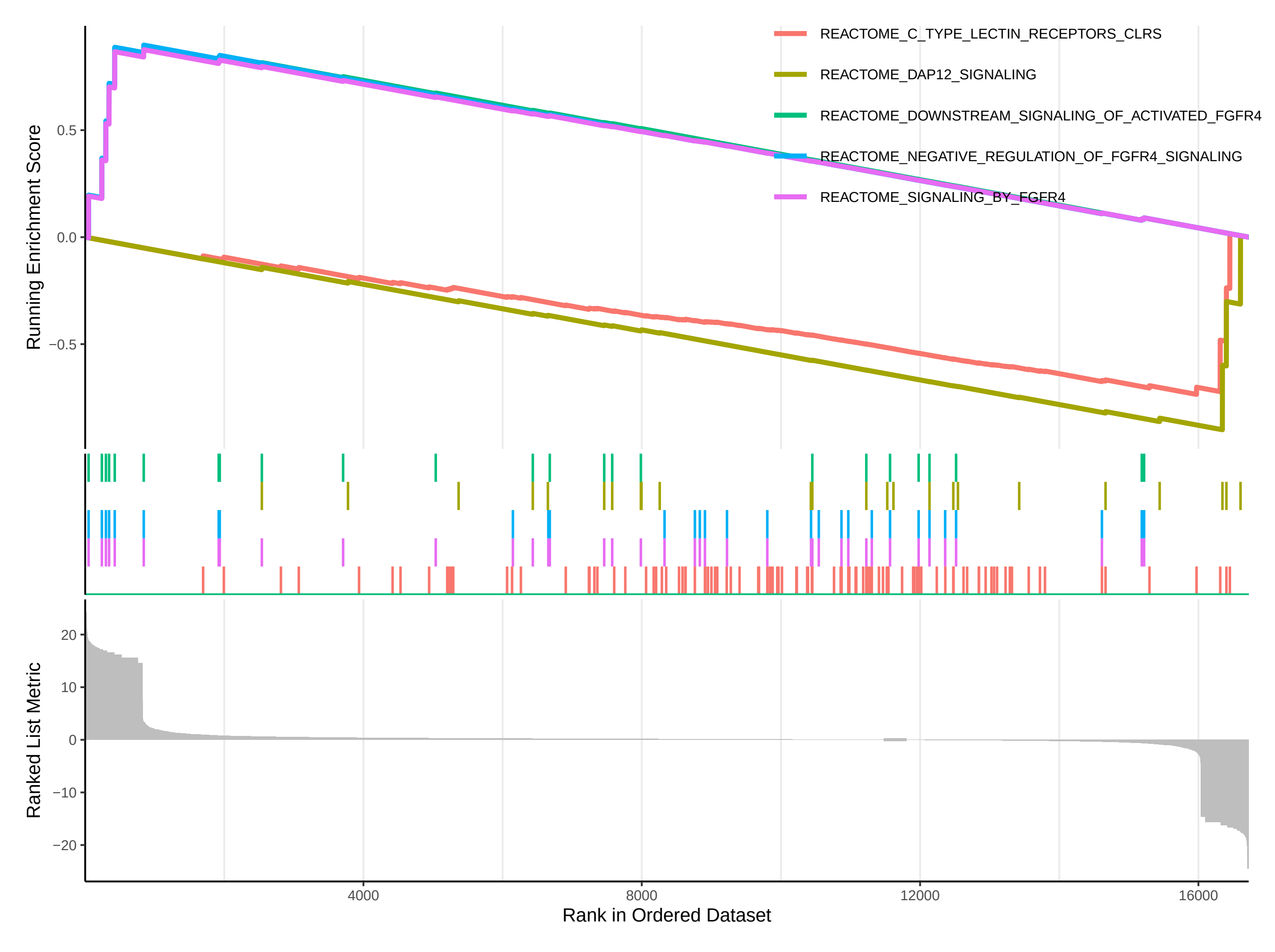
REACTOME_C_TYPE_LECTIN_RECEPTORS_CLRS
REACTOME_DAP12_SIGNALING
REACTOME_DOWNSTREAM_SIGNALING_OF_ACTIVATED_FGFR4
REACTOME_NEGATIVE_REGULATION_OF_FGFR4_SIGNALING
REACTOME_SIGNALING_BY_FGFR4
Running Enrichment Score
0.5
0.0
−0.5
Ranked List Metric
20
10
0
−10
−20
4000
8000
12000
16000
Rank in Ordered Dataset
