# Supplementary material for: Mechanistic Study of Hypoxia-Mediated Regulation of Osteoblast Senescence via ATP6V1A-Dependent Modulation of Metabolic Remodeling
Source: Biology (Basel). 2025 Dec 18;14(12):1801. doi: 10.3390/biology14121801 (PMC12731071; doi:10.3390/biology14121801)
Supplement: Supplementary file 1 [file biology-14-01801-s001.zip › Supplementary Figure4.pdf]

Figure1：

P53：

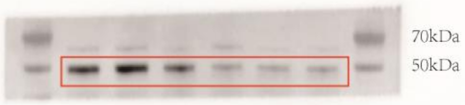

P21：

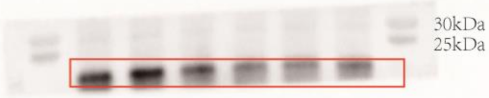

β-actin:

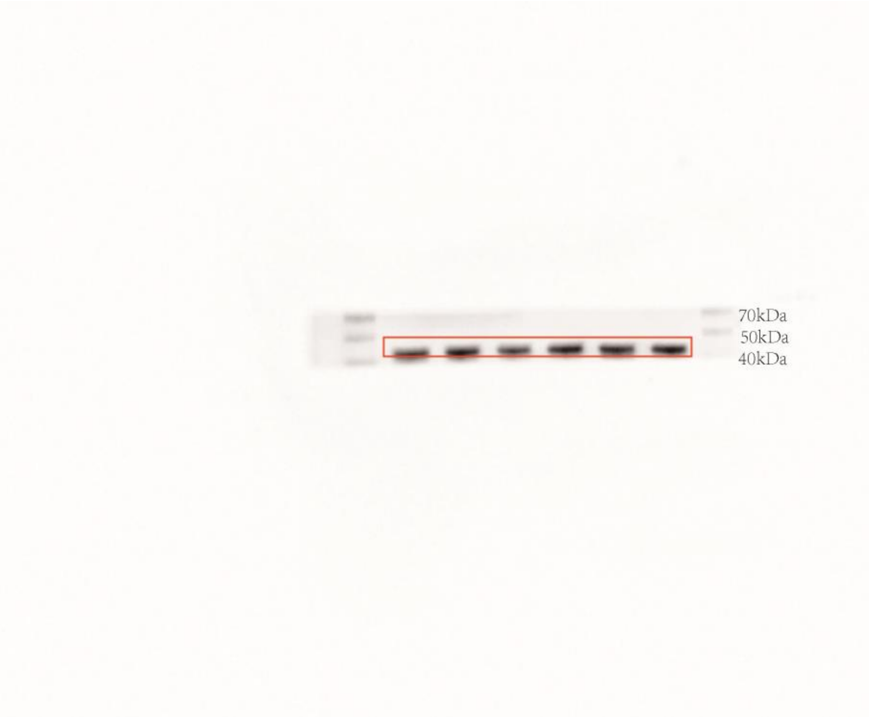

Figure2:

HK2:

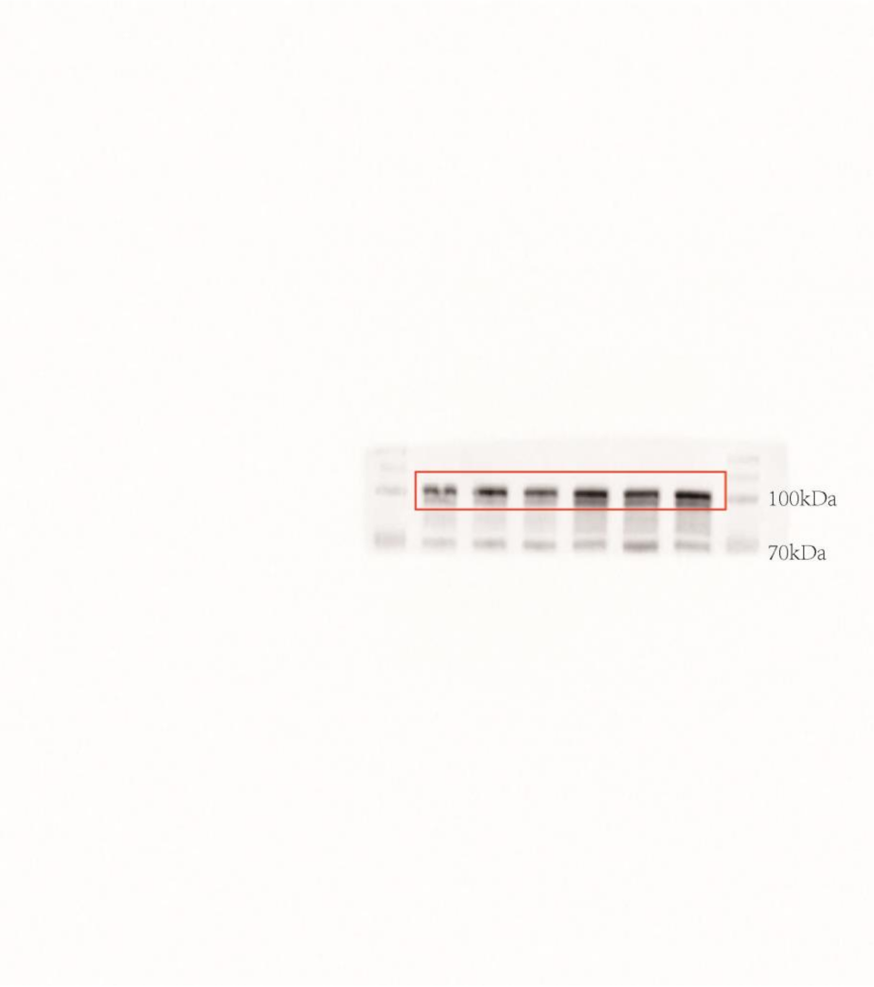

LDH:

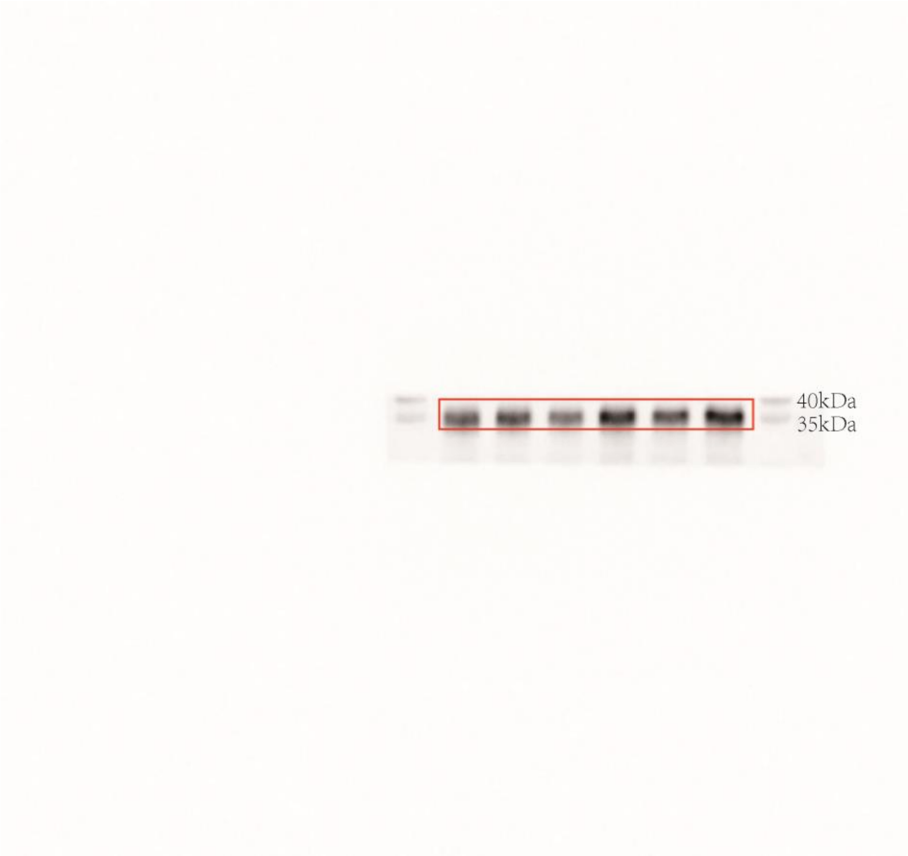

β-actin：

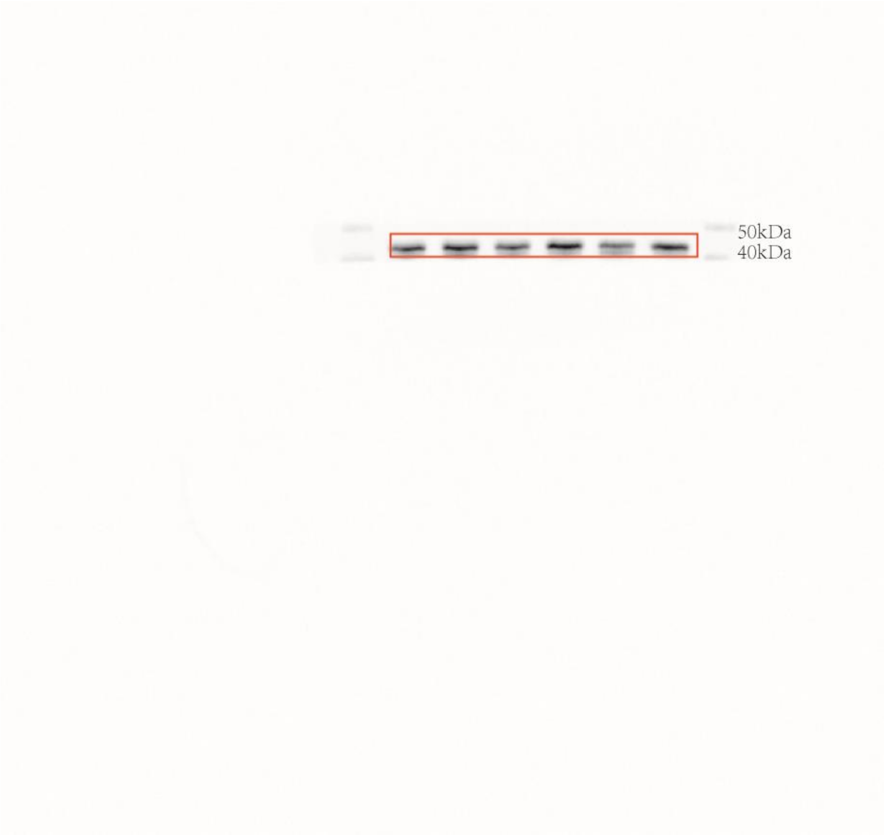

Figure4：

β-actin：

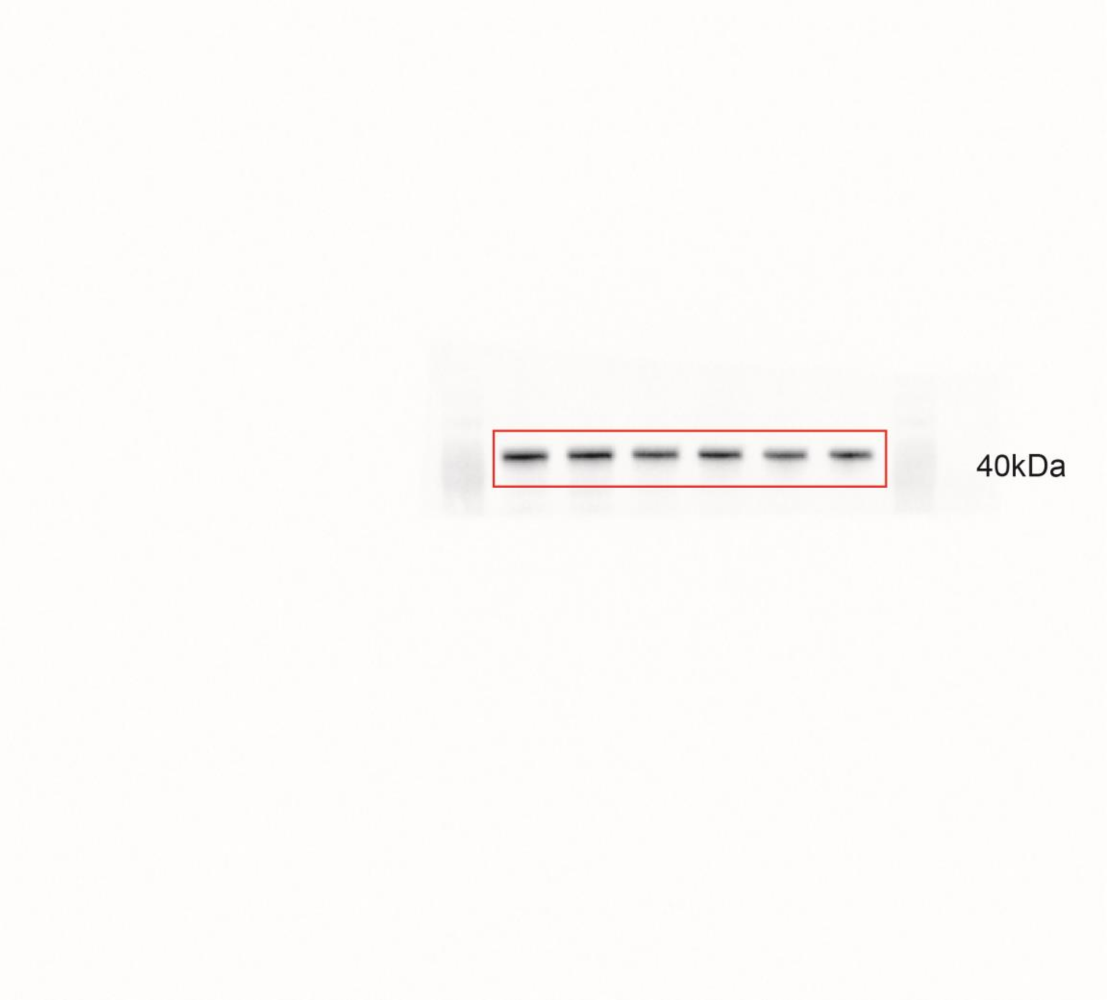

Atp6v1a：

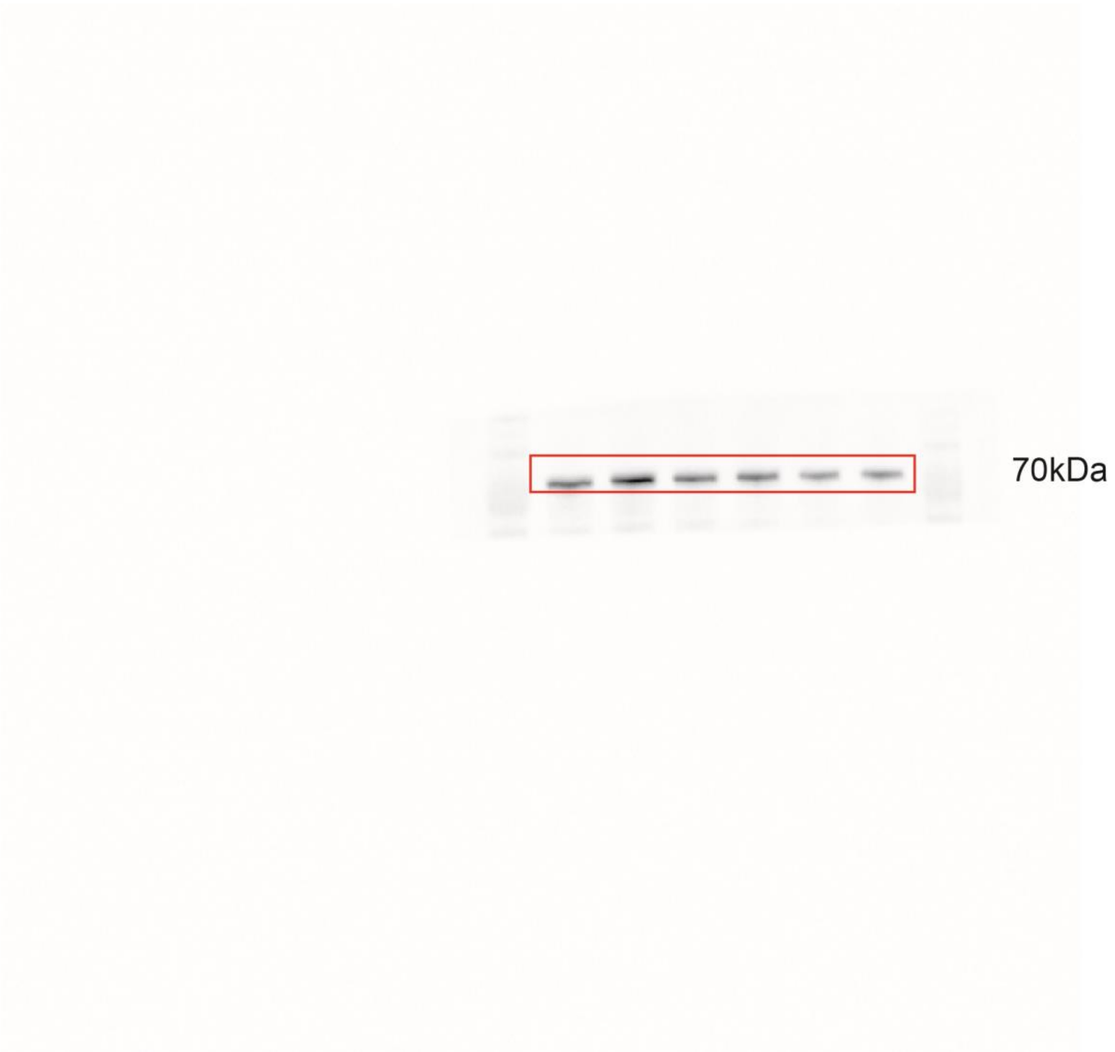

Figure5:

P21:

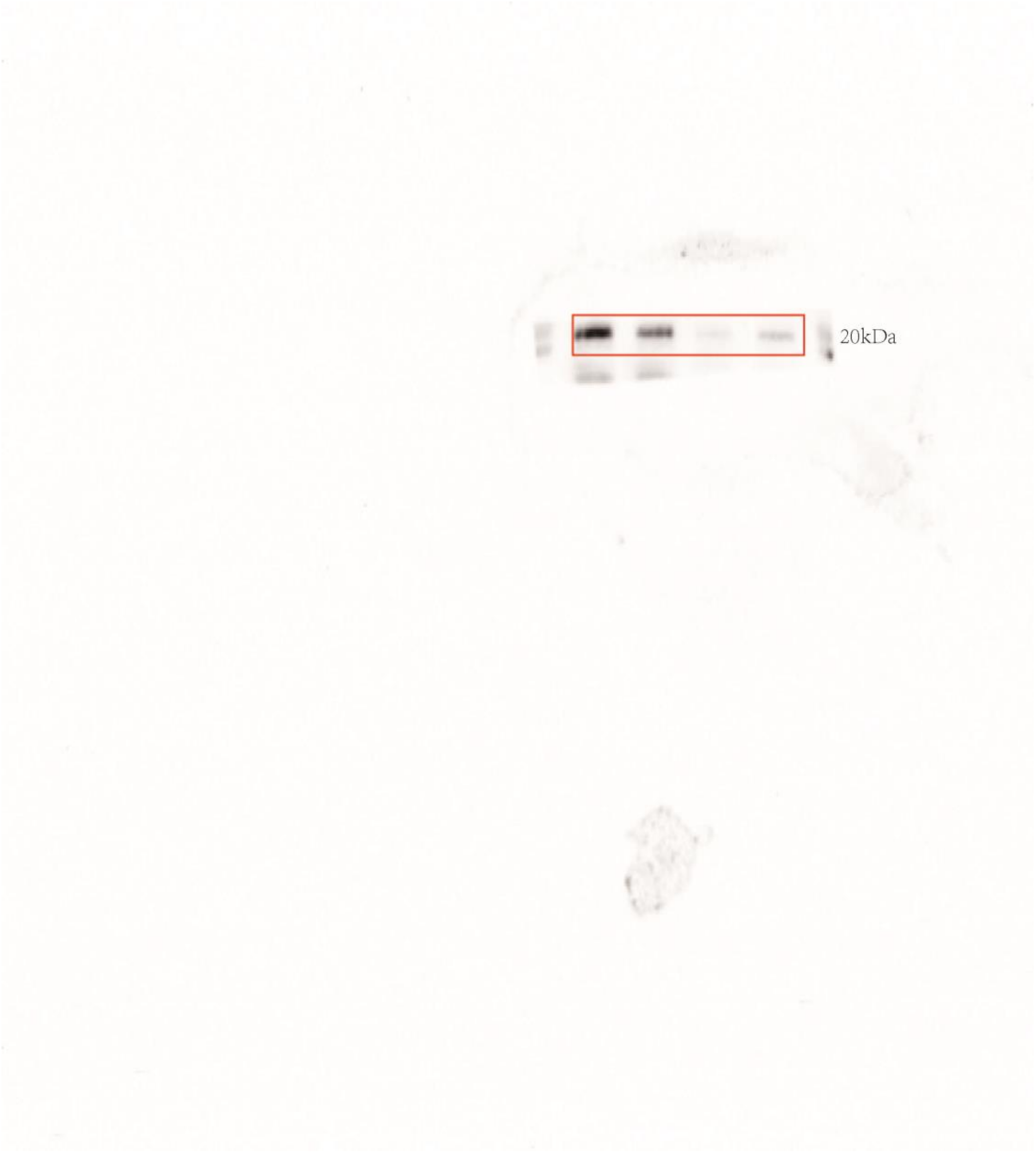

β-actin:

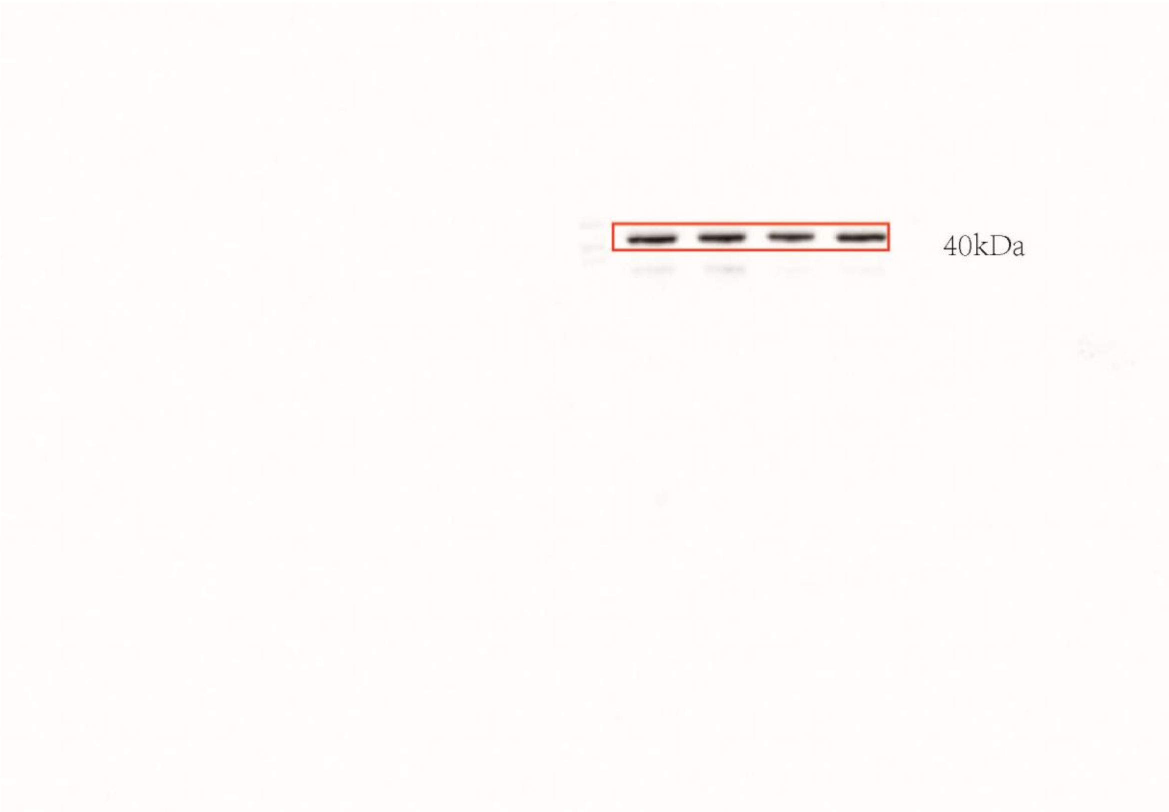

Figure S1

Atp6v1a：

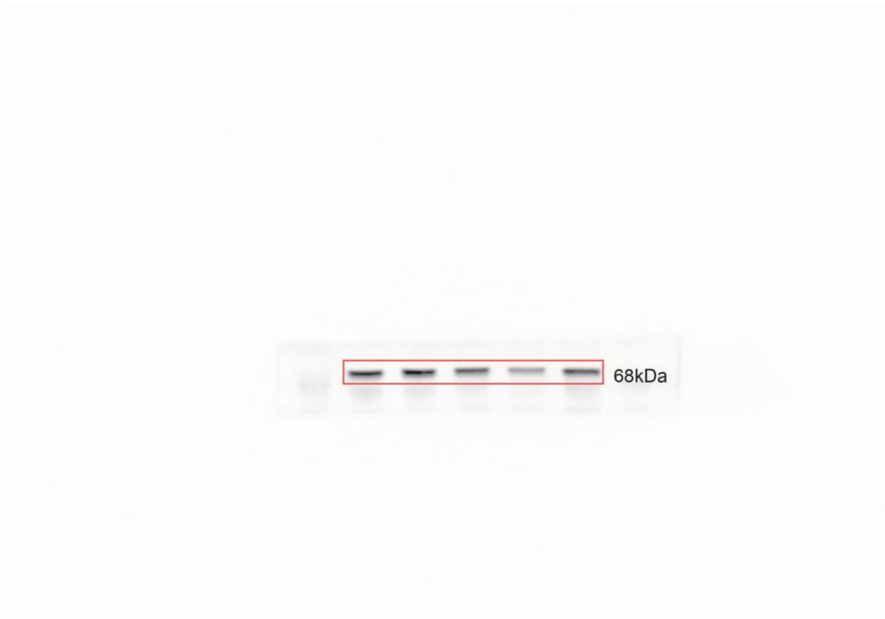

β-actin

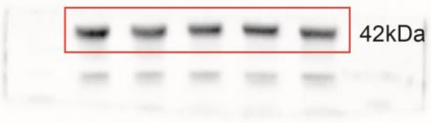
42kDa
