# Supplementary figures and images for: Mechanistic Study of Hypoxia-Mediated Regulation of Osteoblast Senescence via ATP6V1A-Dependent Modulation of Metabolic Remodeling
Source: Biology (Basel). 2025 Dec 18;14(12):1801. doi: 10.3390/biology14121801 (PMC12731071; doi:10.3390/biology14121801)

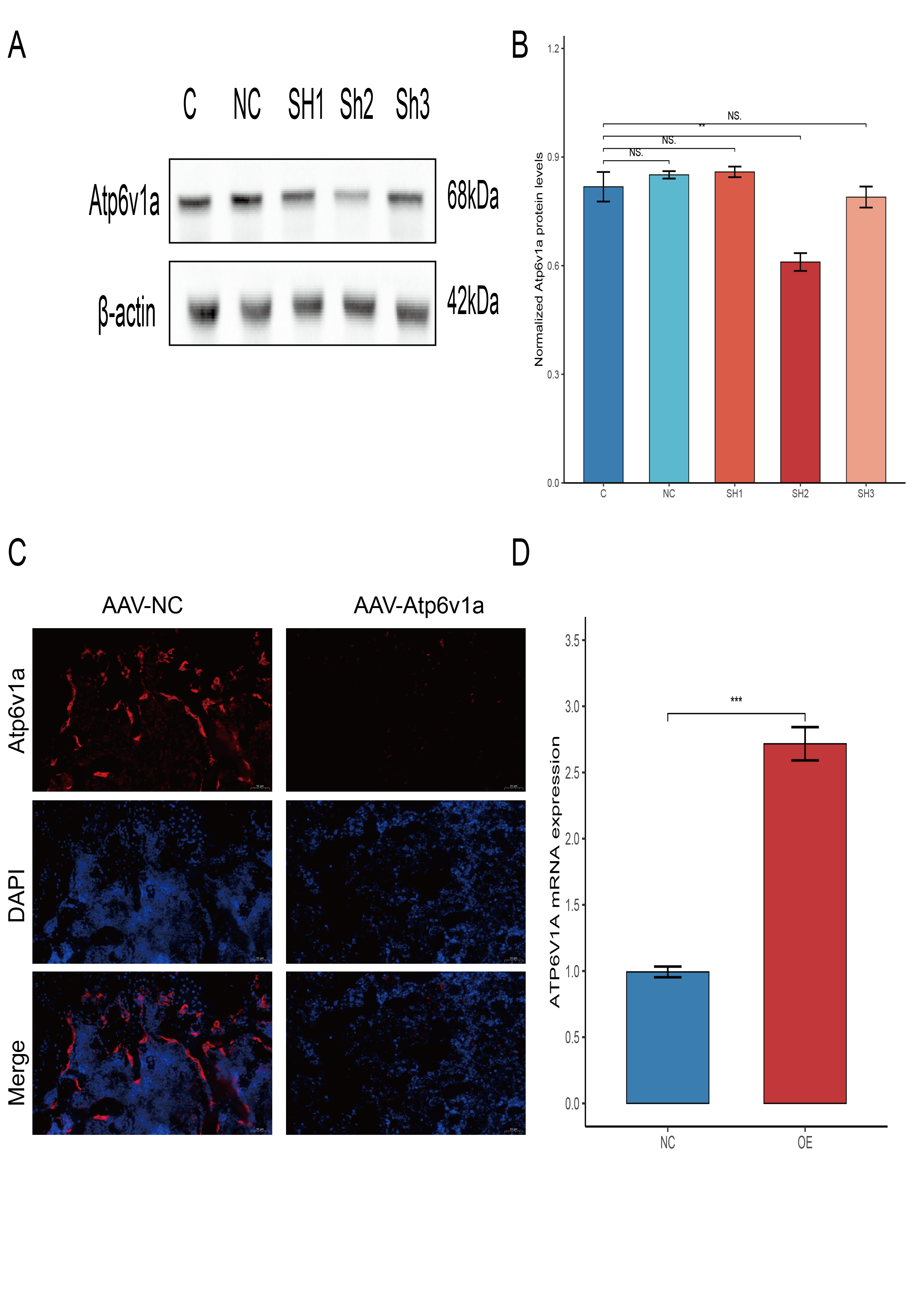

Supplement: Supplementary file 1 [file biology-14-01801-s001.zip › Supplementary Figure1.tif]

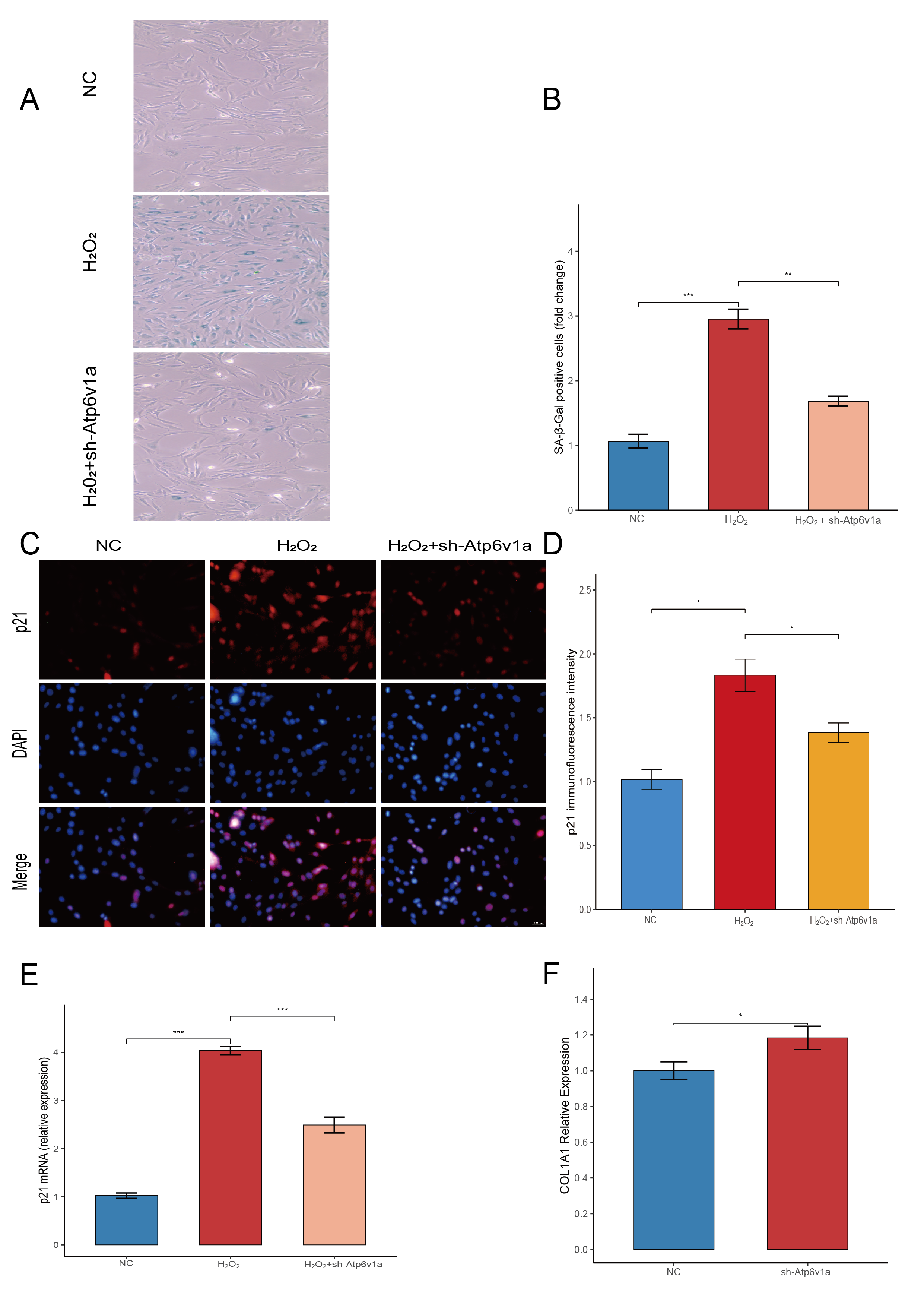

Supplement: Supplementary file 1 [file biology-14-01801-s001.zip › Supplementary Figure3.tif]
