# Supplementary material for: Mechanistic Study of Hypoxia-Mediated Regulation of Osteoblast Senescence via ATP6V1A-Dependent Modulation of Metabolic Remodeling
Source: Biology (Basel). 2025 Dec 18;14(12):1801. doi: 10.3390/biology14121801 (PMC12731071; doi:10.3390/biology14121801)
Supplement: Supplementary file 1 [file biology-14-01801-s001.zip › Supplementary Information Legends.doc]

**Supplementary Figure 1. Validation of ATP6V1A knockdown and overexpression in vitro and in vivo.**

(A) Western blot showing reduced ATP6V1A protein levels after shRNA transduction in MC3T3-E1 cells. (B) Quantified Western blot analysis showing that SH2 achieves the greatest reduction in ATP6V1A levels (n = 3). (C) Immunofluorescence of femoral bone confirming decreased ATP6V1A expression in AAV-treated mice (n=6). (D) qPCR confirming effective ATP6V1A overexpression in MC3T3-E1 cells (n=3). *P < 0.05, P < 0.01, *P < 0.001, ****P < 0.0001, ns = not significant.

**Supplementary Figure 2. Gene Set Enrichment Analysis (GSEA) of ATP6V1A-associated transcriptional programs.**

GSEA based on transcriptomic profiling reveals the signaling pathways enriched in ATP6V1A.

**Supplementary Figure 3. Validation of ATP6V1A knockdown in an H₂O₂-induced senescence model.** (A) Representative SA-β-gal staining images of NC, H₂O₂-treated, and H₂O₂ + sh-Atp6v1a groups. (B) Quantification of SA-β-gal–positive cells showing increased senescence after H₂O₂ stimulation and attenuation upon ATP6V1A knockdown (n=3). (C) p21 immunofluorescence staining demonstrating strong induction of p21 by H₂O₂ and partial reversal by sh-Atp6v1a. (D) Quantification of p21 fluorescence intensity (n=3). (E) qPCR analysis of p21 mRNA expression (n=3). (F) qPCR results showing mild upregulation of COL1A1 following ATP6V1A knockdown (n=3). *P < 0.05, P < 0.01, *P < 0.001, ****P < 0.0001, ns = not significant.

**Supplementary Figure 4: Supplementary Information-Uncropped Immunoblots.**

**Supplementary** Tables1. Primers used for qPCR

**Supplementary** Tables2 V1 subunit–related genes

**Supplementary** Tables3 DEGs identified
