# Supplementary material for: Mechanistic Study of Hypoxia-Mediated Regulation of Osteoblast Senescence via ATP6V1A-Dependent Modulation of Metabolic Remodeling
Source: Biology (Basel). 2025 Dec 18;14(12):1801. doi: 10.3390/biology14121801 (PMC12731071; doi:10.3390/biology14121801)
Supplement: Supplementary file 1 [file biology-14-01801-s001.zip › Supplementary Tables1.doc]

Table S1. Primers used for qPCR

| Target gene | Forward | Reverse |
| --- | --- | --- |
| p53 | ACCGCCGACCTATCCTTACCATC | GGCACAAACACGAACCTCAAAGC |
| p21 | CCTTGTCGCTGTCTTGCACTCTG | GCTGGTCTGCCTCCGTTTTCG |
| β-actin | GATGGTGGGAATGGGTCAGAAGG | TTGTAGAAGGTGTGGTGCCAGATC |
| Col1a1 | CCCTGGTCСCCTGGGAAATG | GGACCTTTGCСССТTССТТТ |
